# Thoracic actinomycosis in a bronchopulmonary sequestration: An extremely rare encounter

**DOI:** 10.5339/qmj.2024.qitc.10

**Published:** 2024-03-25

**Authors:** Aasir M. Suliman, Ahmed Alsayed, Sarah Obiedat, Ehab Massad, Irfan Ul Haq

**Affiliations:** 1 Pulmonology Department, Hamad General Hospital, Hamad Medical Corporation, Doha, Qatar Email: asuliman4@hamad.qa; 2 Laboratory Medicine and Pathology Department, Hamad General Hospital, Hamad Medical Corporation, Doha, Qatar; 3 Thoracic Surgery Department, Hamad General Hospital, Hamad Medical Corporation, Doha, Qatar

**Keywords:** Actinomycosis, Bronchopulmonary sequestration, congenital lung anomaly

## Background

Bronchopulmonary sequestration (BPS) is a rare congenital malformation of the lower respiratory tract that consists of a nonfunctioning mass of lung tissue supplied by anomalous vessels.^1^ Pulmonary actinomycosis is an uncommon infection that remains a diagnostic challenge due to its non-specific symptoms that may mimic other chronic pulmonary infections and malignancies.^2^ BPS is seldom associated with superadded Actinomyces infection, further complicating the clinical presentation.

## Case Presentation

**Figure 1.**
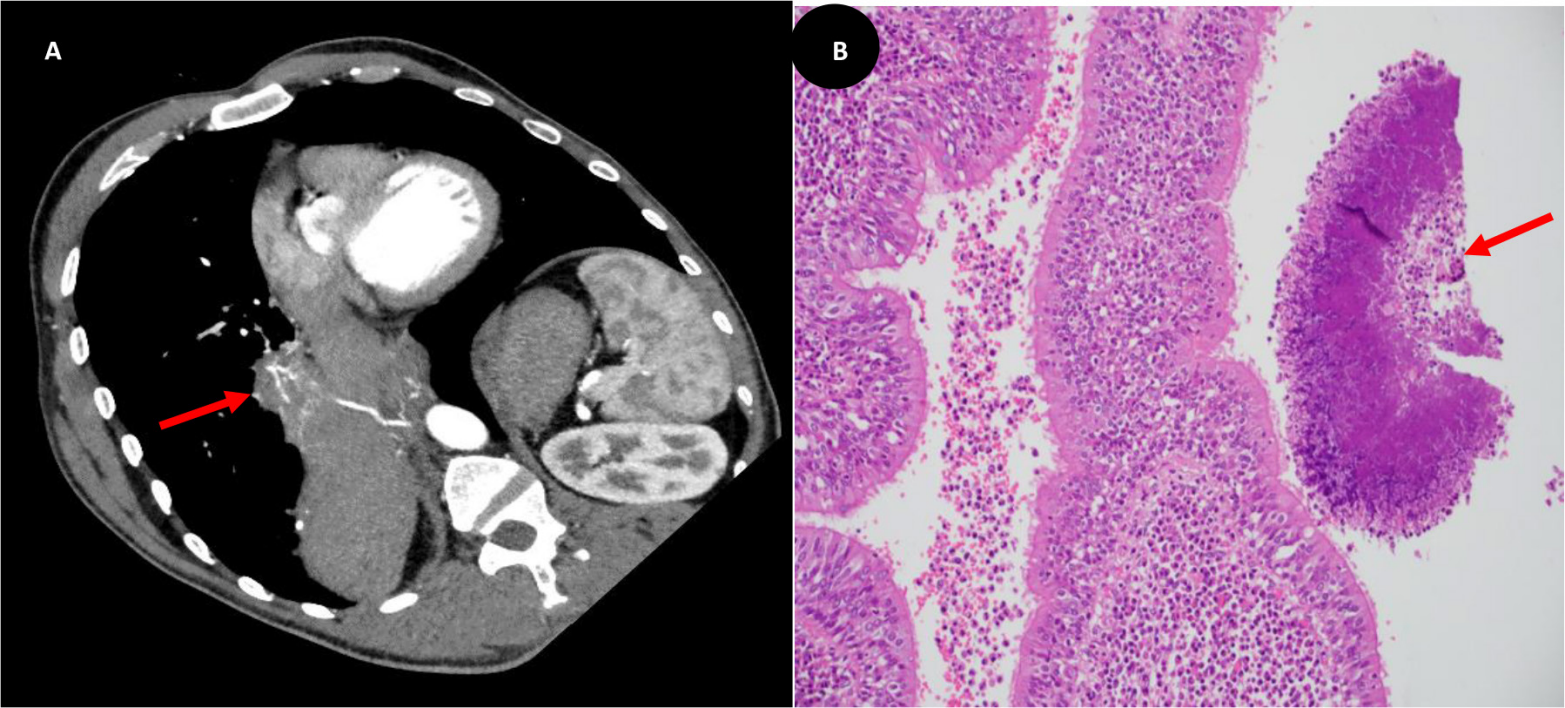
An oblique reconstruction computed tomography view demonstrates a large irregular heterogenous lesion in the inferior and medial aspect of the right lower lobe (red arrow), with an anomalous arterial supply (A). A medium power microscopic view of a colony of Actinomyces species showing filaments radiating from a central mass within a dilated and inflamed bronchiole (red arrow) (B).

We present the case of a 48-year-old man with a two-month history of productive cough and hemoptysis, and who experienced massive hemoptysis during admission. Urgent chest computed tomography (CT) revealed an intralobular BPS, prompting immediate surgical intervention (Figure 1A). Histopathological examination confirmed the diagnosis of BPS, revealing an underlying actinomycosis as an unusual complicating factor (Figure 1B). A prolonged course of antibiotics was initiated, resulting in a notable clinical improvement observed during routine follow-up.

## Conclusion

This case report highlights an unusual association between BPS and pulmonary actinomycosis. Early identification of this complex clinical entity can be challenging due to their similar nonspecific clinical presentation, with a potential clue being the suboptimal response to standard antimicrobials. Accurate identification of the causative organism, particularly atypical pathogens such as Actinomyces, is crucial in BPS patients, given their higher incidence of infection.

## Conflict of Interest

The authors declare that they have no known competing financial or personal interests.
